# Inhibition of Podocytes DPP4 Activity Is a Potential Mechanism of Lobeliae Chinensis Herba in Treating Diabetic Kidney Disease

**DOI:** 10.3389/fphar.2021.779652

**Published:** 2021-12-07

**Authors:** Xinyu Wang, Jiaqing Xiang, Guixiao Huang, Lin Kang, Guangyan Yang, Han Wu, Kewei Jiang, Zhen Liang, Shu Yang

**Affiliations:** ^1^ Department of Geriatrics, The Second Clinical Medical College of Jinan University, Shenzhen People’s Hospital, Jinan University, Shenzhen, China; ^2^ The Third Affiliated Hospital of Shenzhen University, Shenzhen, China; ^3^ Department of Endocrinology, The Second Clinical Medical College of Jinan University, Shenzhen People’s Hospital, Jinan University, Shenzhen, China

**Keywords:** diabetic kidney disease, dipeptidyl peptidase-4, lobeliae chinensis herba, network pharmacology, podocytes, single-cell sequencing

## Abstract

Diabetic kidney disease (DKD) is the leading cause of end-stage renal disease and has become a serious public health problem worldwide. Dipeptidyl peptidase-4 (DPP4) inhibitors, an emerging drug for the treatment of diabetes, have been found to have renoprotective effects in addition to glucose-lowering effects and therefore have the potential to be a treatment modality for DKD. Lobeliae Chinensis Herba (LCH), a traditional Chinese herb widely used in the treatment of diabetes, has recently been found to have a hypoglycaemic mechanism related to the inhibition of DPP4. Firstly, analysis of single-cell sequencing data from mouse kidneys in the National Center for Biotechnology Information (NCBI) database revealed that DPP4 was specifically upregulated in DKD podocytes and was associated with podocyte proliferation. Subsequently, the network pharmacology approach was applied to the screening of compounds. Twelve LCH active ingredients targeting DPP4 were extracted from the Traditional Chinese Medicine System Pharmacology (TCMSP) database. In addition, these 12 compounds and DPP4 were molecularly docked to predict the probability of them affecting DPP4 activity. *In vitro*, Quercetin, Methyl rosmarinate, Kaempferol, Diosmetin and Acacetin were demonstrated to retard podocyte proliferation by inhibiting DPP4 activity and were the top five compounds predicted by molecular docking to be the most likely to affect DPP4 activity. The half maximal inhibitory concentration (IC_50_) of the five compounds for DPP4 activity were as follows. Acacetin Log IC_50_ = −8.349, 95%CI (−9.266, −7.265), Diosmtrin Log IC_50_ = −8.419, 95%CI (−8.889, −7.950), Log IC_50_ = −8.349, 95%CI (−9.266, −7.265), Methyl rosmarinate Log IC_50_ = −8.415, 95%CI (−8.751, −8.085), Kaempferol Log IC_50_ = −8.297, 95%CI (−9.001, −7.615), Quercetin Log IC_50_ = −8.864, 95%CI (−9.107, −8.615). Finally, Quercetin, Methyl rosmarinate, Kaempferol, Diosmetin and Acacetin qualified for pharmacokinetic and drug similarity screening and have the potential to be the most promising oral agents for the treatment of DKD.

## Introduction

Diabetic kidney disease (DKD) is one of the major devastating complications of diabetes, with an increasing global prevalence and a huge health and economic burden (Forbes et al., 2008; Cho et al., 2018). The pathological changes characteristic of DKD include thickened glomerular basement membrane, podocyte injury, dilated mesangial matrix, and loss of glomerular endothelial foramen (Reidy et al., 2014). The disruption of the glomerular filtration barrier due to these types of cell damage, particularly damage to the podocytes, is a key factor in the progression of DKD (Podgórski et al., 2019; Fu et al., 2020). So far, the main therapy for DKD is still blood glucose, weight and blood pressure control, and it lacks specific treatment.

Dipeptidyl peptidase-4 (DPP4) is an inherent type II transmembrane glycoprotein and serine exopeptidase that is highly involved in glucose and insulin metabolism (Lambeir et al., 2003; Röhrborn et al., 2014). DPP4 inhibitors, a new type of glucose-lowering drug, have a renoprotective effect in addition to the glucose-lowering effect (Hasan and Hocher, 2017). DPP4 inhibitors can achieve renal protection through a variety of pathways. Kanasaki’s study demonstrated that linagliptin, a DPP4 inhibitor, ameliorated renal fibrosis in DKD mice by inhibiting the conversion of endothelial cells to mesenchymal cells (Kanasaki et al., 2014). Jung et al. demonstrated that DPP4 inhibitors improved urinary protein by reducing podocyte apoptosis in DKD mice (Jung et al., 2015). CARMELINA, TECOS and SAVOR-TIMI53, three large-scale randomised controlled trials (RCT), all concluded that DPP4 inhibitors significantly reduced urinary protein (Cornel et al., 2016; Mosenzon et al., 2017; Rosenstock et al., 2019). Such shreds of evidence suggest that DPP4 inhibitors will be an important drug for the treatment of DKD. However, the American Diabetes Association has not yet recommended DPP4 inhibitors as the first choice of hypoglycaemic agent for patients with DKD (Selby and Taal, 2020).

Many herbs have been discovered to inhibit the activity of DPP4. Schizandra Chinensis Baill, Coptis Chinensis, *Psidium guajava* L. leaves and *Morus alba* L. leaves were shown to inhibit the activity of DPP4 *in vitro* (Wang and Chiang, 2012). The anti-hyperglycaemic effect of Stevia rebaudiana was associated with the attenuation of DPP4 activity (Abdel-Aal et al., 2021). DPP4 inhibition by *Terminalia arjuna* was comparable to vildagliptin (Mohanty et al., 2019). Therefore, the search for DPP4 inhibitors from natural products is a viable approach. As a traditional Chinese herbal, Lobeliae Chinensis Herba (LCH) is widely used in the treatment of diabetes. LCH is belonging to the family Campanulaceae and has recently been identified as a potential target of DPP4 for the treatment of diabetes through network pharmacology (Ge et al., 2020). Consequently, the active constituents of the LCH have the potential to treat DKD by inhibiting DPP4.

Single-cell sequencing optimises traditional sequencing methods by identifying the expression of target genes in specific cell clusters (Fu et al., 2019). Although DPP4 is involved in the process of DKD, the most critical cell clusters remain uncertain. Therefore, this study used network pharmacology combined with single-cell sequencing to identify drug targets at the single-cell level.

## Materials and Methods

### Single-Cell Sequencing Data Analysis

The Single-cell profiling of kidney cells sequencing data was acquired from the Gene Expression Omnibus (GEO) GSE127235 dataset of the National Center for Biotechnology Information (NCBI; https://www.ncbi.nlm.nih.gov/gds). Single-cell RNA-seq was done on the Fluidigm C1 800-cell HT platform (v2), and sequenced paired-end on the Illumina NextSeq 500 platform (Fu et al., 2019). The Seurat package of R software was applied uniform manifold approximation and projection (UMAP) for single-cell sequencing data dimensionality reduction visualization (Becht et al., 2018; Kang et al., 2018). Changes in variation in expression of differentially expressed genes between groups were analysed by log(2)-fold changes (Zhao et al., 2019).

### Construction of PPI Network

The protein-protein interaction (PPI) network of genes was constructed by the STRING web tool (https://string-db.org/). The STRING database aggregates all available information about protein interactions, and complement these with computational predictions (Szklarczyk et al., 2019). CYTOSCAPE, a software for proteomic data network analysis and visualization, was used to optimize the results of STRING analysis (Doncheva et al., 2019).

### Enrichment Analysis

ENRICHR, an enrichment analysis web tool, was used for gene signalling pathways and ontology analysis (https://maayanlab.cloud/Enrichr/) (Chen et al., 2013). The ggplot2 and ggpubr package of R software were used for visualization of enrichment results (Ito and Murphy, 2013; Whitehead et al., 2019), including terms, gene ratio, gene counts, false discovery rate (FDR), etc.

### Screening of Components of LCH

The compounds of LCH were searched through the TCMSP database (http://tcmspw.com/index.php). TCSMP is a pharmacological platform that captures the relationships between drugs, target genes and diseases (Ru et al., 2014). Subsequently, the compounds targeting DPP4 were further extracted and satisfied the screening conditions of support vector machine (SVM) and random forest (RF) scores ≥0.8 and 0.7, respectively (Ballester and Mitchell, 2010; Leong et al., 2017).

### Molecular Docking

Discovery Studio (DS) was used for the molecular docking of DPP4 and compounds. DS 2019 version is a molecular modelling software for protein structure studies and drug discovery (Zhang et al., 2020). The structure of small molecule compounds and DPP4 were downloaded from TCSMP and Protein Data Bank (PDB) database (https://www.rcsb.org) (Karuppasamy et al., 2020), respectively. First, the compounds were used for ligand preparation, a method to remove duplicates, enumerating isomers and tautomers, and generating 3D conformations. Next, a series of preparations were also applied to the protein receptor, including removing water molecules, adding hydrogen atoms, setting up active pockets, etc. Finally, CDocker was used for molecular docking, an algorithm that allows precise docking of any number of ligands to a single protein receptor (Wu et al., 2003). -CDOCKER Interaction Energy (CIE) reflects the ability of ligands and receptors to interact in molecular docking.

### Pharmacokinetic and Druglikeness Analysis

SwissADME (http://www.swissadme.ch), a network analysis tool, is designed to assess the pharmacokinetics and drug-likeness of small molecules (Daina et al., 2017a). By uploading the SMILES list of small molecule compounds, both relevant analytical results become available. The assessment of drug-likeness characterisation was based on five rule-based filters from pharmaceutical companies, Lipinski (Pfizer), Ghose (Amgen), Veber (GSK), Egan (Pharmacia) and Muegge (Bayer). The pharmacokinetic analysis includes principally gastrointestinal (GI) adsorption, blood-brain barrier (BBB) penetration, permeability glycoproteins(P-gp) substrates and cytochrome p450 (CYP) inhibitors. The absorption of small molecules in the human gastrointestinal and penetration of the blood-brain barrier was predicted based on the BOILED-Egg model (Daina and Zoete, 2016). Screening of substrates of P-gp or isozyme inhibitors of CYP based on vector machine algorithm (SVM) for large data sets of known substrate/non-substrate or inhibitor/non-inhibitor (Cortes and Vapnik, 1995).

### Cell Cultures

Immortalized human podocyte and mesangial cell lines were used in this study (Lee, 1995; Ni et al., 2012). Immortalized cells were obtained from primary podocytes and mesangial cells by infection with a hybrid Adeno5/SV40 virus. Cells were incubated in a humidified incubator at 37°C, 5% CO_2_ and in DMEM supplemented with 10% fetal bovine serum, penicillin G (100 U/ml), streptomycin (100 μg/ml) and l-glutamine (2.0 mM). Cell culture medium was changed on a 2-day cycle.

### DPP4 Actitivty Assay

DPP4 activity (MAK088, Sigma) in cells and in kidney was assessed and calculated by using standard methods according to manufacturers’ instructions.

### Cell Growth Measurement and Cell Cycle Analysis

Cells were cultured in 24-well plates (2 × 10^3^ cells per well) and separately exposed to vectors (control), Acacetin, Kaempferol, Methyl rosmarinate, Quercetin, Diosmin. Cell growth was evaluated in sub-confluent cultures using the 3-(4, 5-dimethyl thiazole-2-yl)-2, 5-diphenyltetrazole bromide (MTT) colourimetric assay; results were confirmed by determining cell density, as previously described (Miglio et al., 2011; Miglio et al., 2012).

The percentage of cells at different stages of the cell cycle is described above (Miglio et al., 2005). In brief, at the end of each treatment, cells were washed with PBS and centrifuged for collection. The cells were re-suspended with cold ethanol (70%) and held at 20°C for at least 24 h. The cells were then washed twice with PBS and treated with RNase (0.5 mg/ml, final concentration) (1 h, 37°C). Finally, propidium iodide (PI; 50 μg/ml) was added. Analyses were performed on a Beckman DXFLEX Flow Cytometer.

## Result

### Dpp4 Expression Was Specifically Up-Regulated in Podocytes of DKD Mice

Before clustering the single-cell data, quality control was first performed (Supplementary Figures 1, 2). It was found that the amount of gene expression detected was strongly positively correlated with the number of genes detected in the cells, while the amount of gene expression detected in the cells was not correlated with the proportion of mitochondria. Cells with greater than 2,500 and less than 200 genes detected per cell were subsequently filtered out to avoid too many/too few genes. Also, cells with more than 5% mitochondrial occupancy were filtered out.

**FIGURE 1:**
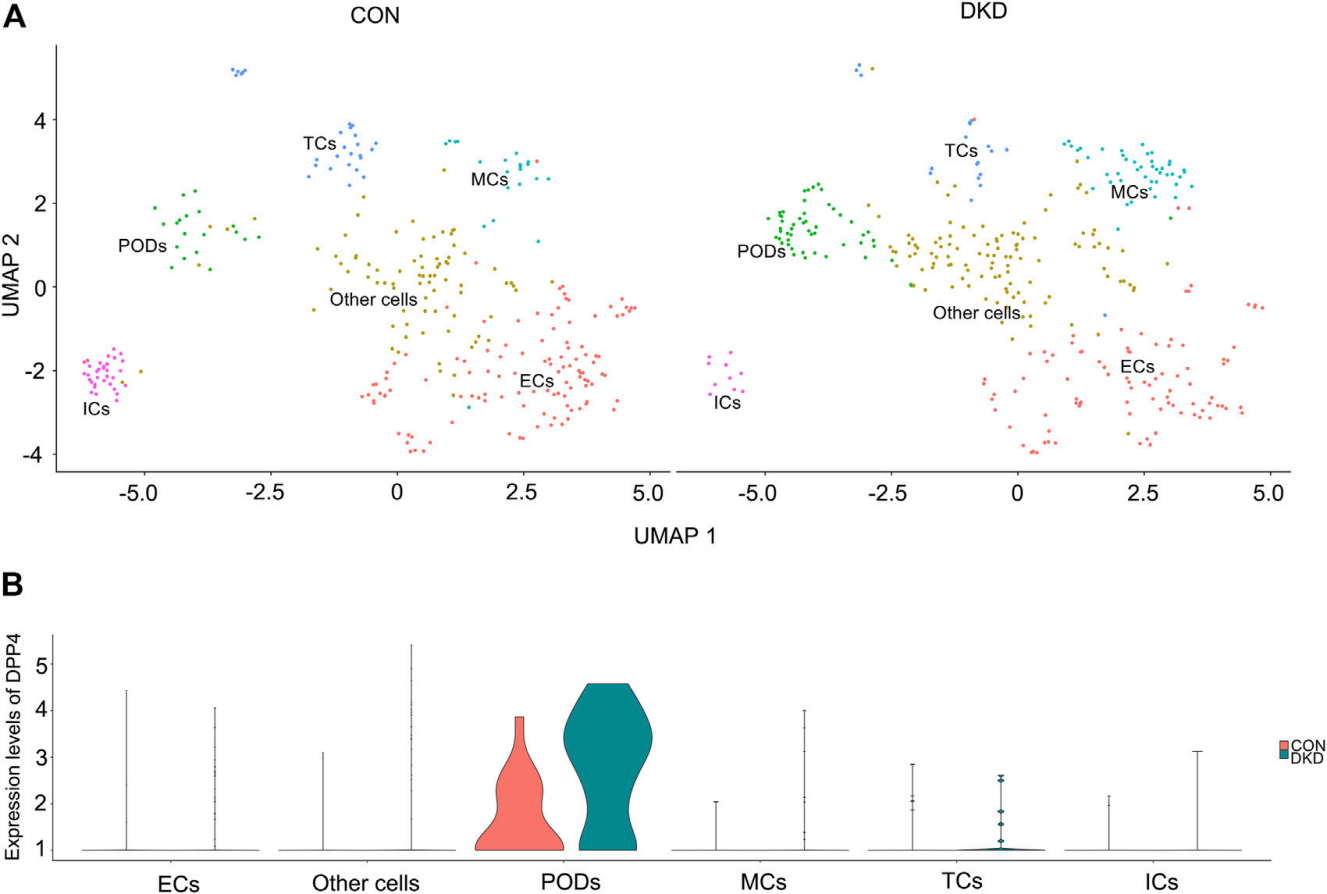
Single-cell sequencing analysis of the mouse control and DKD kidneys. **(A)** UMAP analysis of kidney cells revealed six types of cell clusters in control **(left)** and DKD group **(right)** mice. **(B)** Single-cell sequencing identified significant specific upregulation of Dpp4 in podocytes (*p*-value was calculated by Wilcoxon rank sum test, *p* < 0.001, log_(2)_FC = 1.25). Abbreviation: ECs, endothelial cells; ICs, immune cells; MCs, mesangial cells; PODs, podocytes; TCs, tubular cells.

The kidney cells of DKD mice were divided into six cell clusters by single-cell sequencing, which were endothelial cells, podocytes, mesangial cells, tubular cells, immune cells and other undefined cells, the number of DKD podocytes increased compared to the control group (Figure 1A). Subsequently, Dpp4 expression was found to be significantly up-regulated in DKD podocytes, with no significant difference in other cell clusters (Figure 1B). This is consistent with previous studies reporting increased DPP4 activity in podocytes at DKD status (Kubo et al., 2020). Further, in the DKD model, DPP4 inhibitors were shown to improve podocyte function (Jung et al., 2015; Eun Lee et al., 2016; Kubo et al., 2020).

### Identification of Differential Genes Interacting with *Dpp4* in Podocytes by PPI Network and Their Enrichment Analysis

**FIGURE 2:**
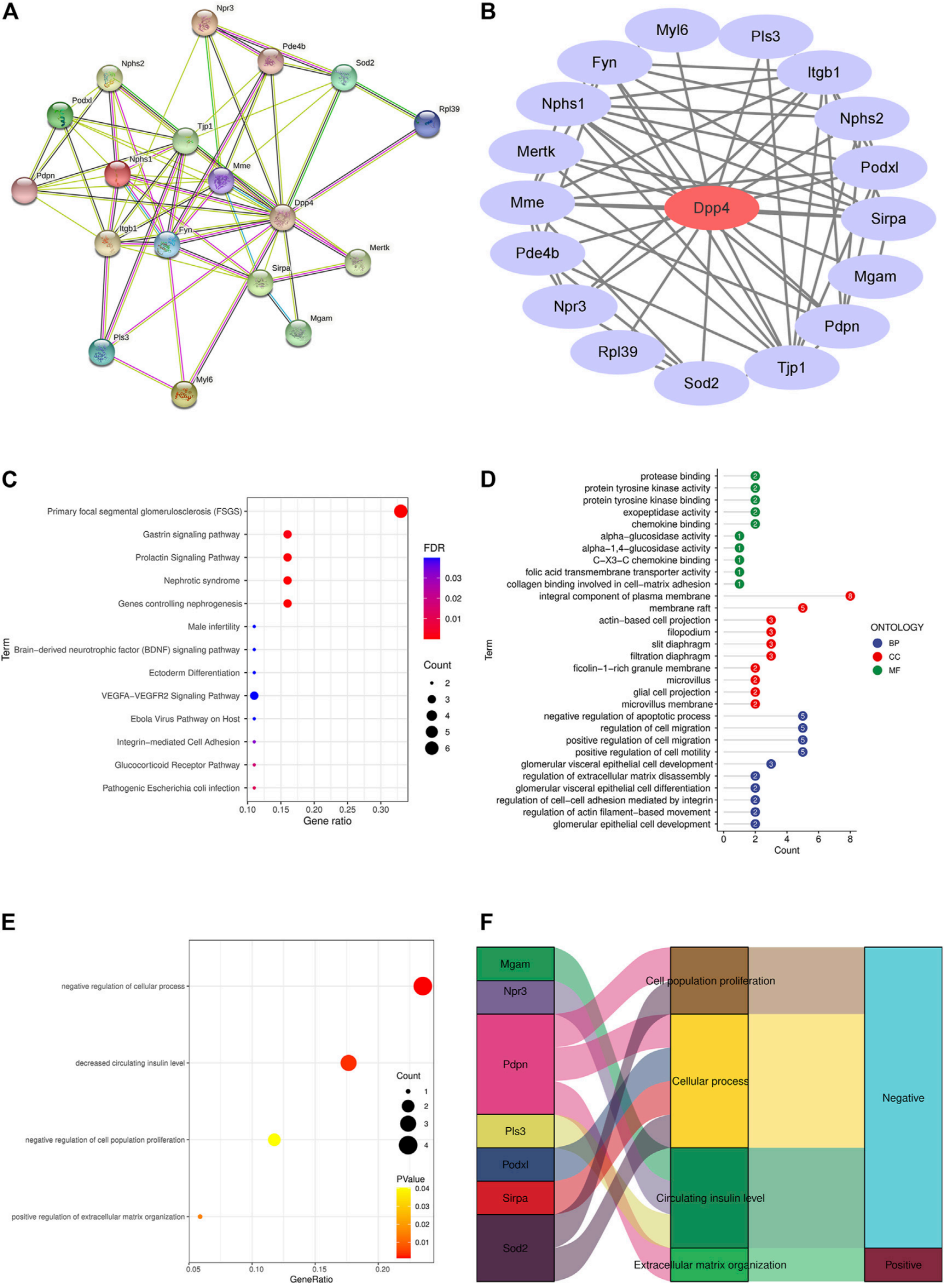
PPI network and enrichment analysis of differential genes associated with Dpp4 in DKD podocytes. **(A)** Proteins predicted by the STRING online tool (https://string-db.org/) to interact with Dpp4 were significantly differentially expressed in DKD (*p* < 0.05). **(B)** The PPI network, optimised by Cytoscape software, highlights the core position of DPP4 (red) with 17 differential genes (purple) at the periphery. **(C)** Enrichment of DKD podocytes differential genes in signalling pathways. **(D)** GO analysis of differential genes in DKD podocytes. **(E)** Screening for genes negatively associated with DPP4 function. **(F)** Sankey diagram showing the genes negatively associated with DPP4 function and the ontological functions in which they are involved.

A total of 17 different genes in DKD mice podocytes were found to be directly related to Dpp4 by the STRING analysis tool, which was Nephrin (Nphs1), Superoxide dismutase (Sod2), Tyrosine-protein kinase Mer (Mertk), Podocalyxin (Podxl), Podocin (Nphs2), Neprilysin (Mme), Podoplanin (Pdpn), Plastin-3 (Pls3), Tyrosine-protein kinase Fyn (Fyn), Atrial natriuretic peptide receptor 3 (Npr3), 60S ribosomal protein L39 (Rpl39), Myosin light polypeptide 6 (Myl6), Phosphodiesterase (Pde4b), Tyrosine-protein phosphatase non-receptor type substrate 1 (Sirpa), Integrin beta-1 (Itgb1), Tight junction protein ZO-1 (Tjp1) and Membrane-bound maltase-glucoamylase (Mgam) (Figures 2A,B). Focal segmental glomerulosclerosis (FSGS), manifested mainly by abnormal proliferation of podocytes (Rosenberg and Kopp, 2017), were found to be the most significantly enriched signalling pathway (Figure 2C). The biological process (BP) was associated with negative regulation of the apoptotic process, which is consistent with our finding of DKD podocyte proliferation in single-cell data. We also found cellular composition (CC) and molecular function (MF) enriched in plasma membrane composition and protease binding, consistent with DPP4 being a plasma membrane-bound form of peptidase (Figure 2D). Ontology analysis in Erichr revealed that *Dpp4* positively regulates cellular processes and cell proliferation, negatively regulates the organization of the extracellular matrix, and is associated with elevated circulating insulin levels. In contrast, *Pdpn* is involved in the positive regulation of extracellular matrix organization, *Pdpn* and *Sod2* negatively regulate cell proliferation, *Podxl, Pdpn, Sirpa, Sod2* are associated with negative regulation of cellular processes, and *Mgam, Npr3, Pls3* may lead to a reduction in circulating insulin levels (Figures 2E,F).

### Screening of Components of LCH Targeting DPP4

**FIGURE 3:**
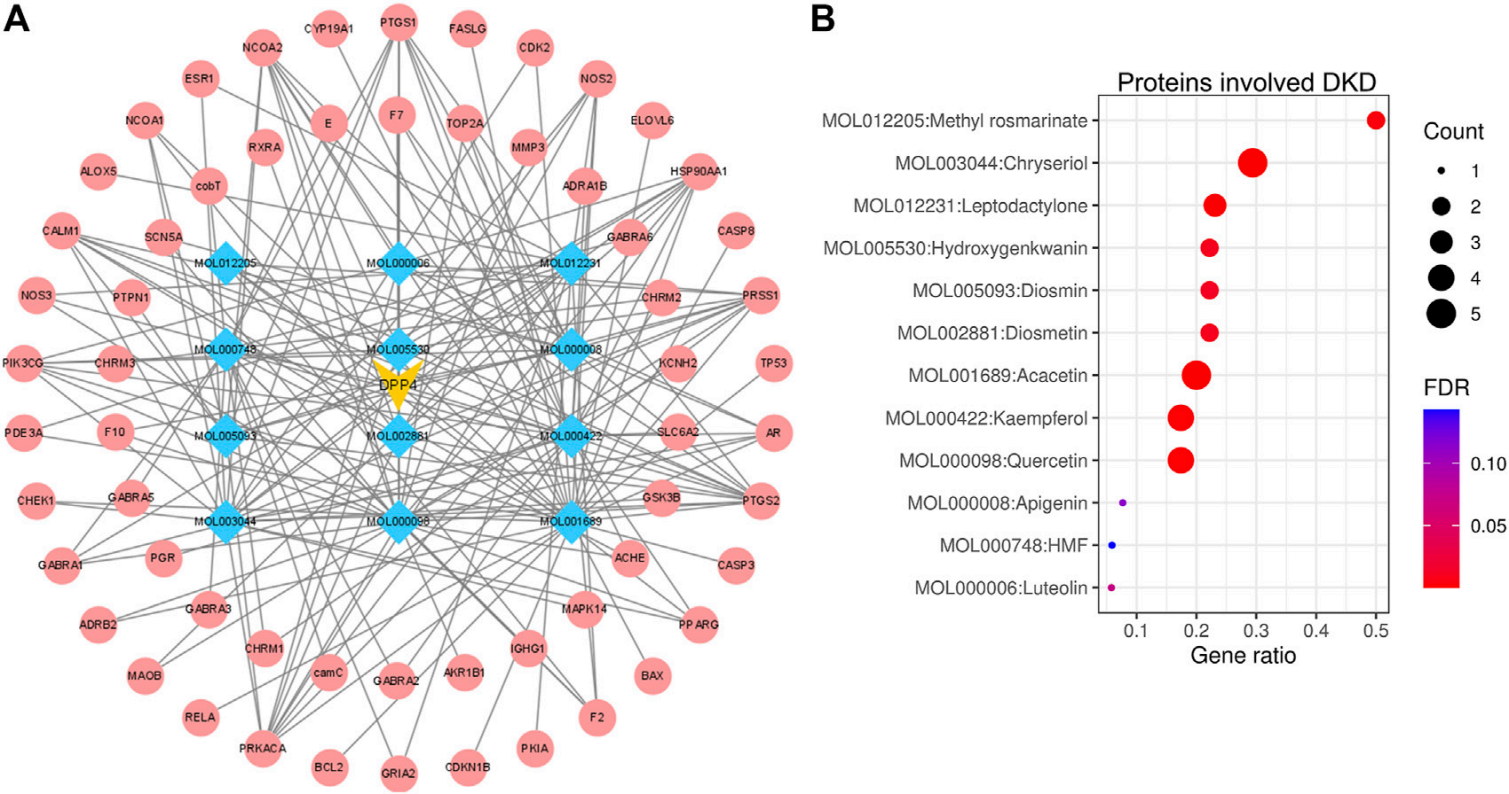
Network construction and signal pathway analysis of DPP4 targeted compounds. **(A)** DPP4 (yellow)-compound (blue)-target genes (red) network. **(B)** The enrichment of 12 compound target genes in DKD-related signalling pathways analysed via the ENRICHR website (https://maayanlab.cloud/Enrichr/). Methyl rosmarinate, leptodactylone, acacetin, diosmetin, chryseriol, kaempferol, diosmin, hydroxygenkwanin and quercetin was significantly enriched in DKD-related signalling pathways (FDR < 0.05). HMF, apigenin and luteolin was not significantly enriched in DKD-related signalling pathways (FDR ≥ 0.05). *p*-value was calculated by Fisher’s exact test or the hypergeometric test. The FDR is an adjusted *p*-value using the Benjamini-Hochberg method for correction for multiple hypotheses testing.

The compounds of LCH were searched through the TCMSP database (http://tcmspw.com/index.php). These compounds were required to meet the screening criteria of support vector machine (SVM) and random forest (RF) scores ≥0.8 and 0.7, respectively. A total of 12 active compounds with DPP4 as the potential target of LCH were screened, which were Methyl rosmarinate, leptodactylone, acacetin, diosmetin, chryseriol, kaempferol, diosmin, hydroxygenkwanin, luteolin, HMF, apigenin and quercetin (Figure 3A; Table 1). Except for Apigenin, HMF and Luteolin, the target genes (exclusion of DPP4) of the remaining nine compounds were significantly enriched in DKD-related signalling pathways (Figure 3B; Table 1).

**TABLE 1:** Active ingredients of LCH with DPP4 as the target gene, including their MOL ID, MOL name, CAS number, molecular structure and target genes involved in the DKD signalling pathway.

MOL ID	MOL name	CAS	Molecular weight	Structure	Target genes related DKD pathway
MOL012205	Methyl rosmarinate	99353-00-1	374.3	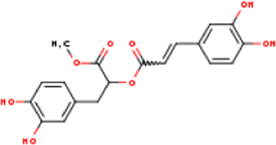	PPARG; PTGS2
MOL012231	Leptodactylone	61899-44-3	222.1	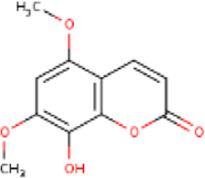	NOS2; NOS3; PTGS2
MOL001689	Acacetin	480-44-4	284.2	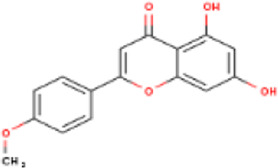	CDKN1B; NOS2; CDK2; PTGS2; TP53
MOL002881	Diosmetin	520-34-3	300.2	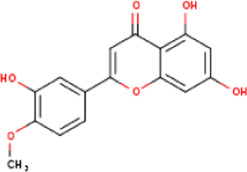	NOS2; PTGS2
MOL003044	Chryseriol	491-71-4	300.2	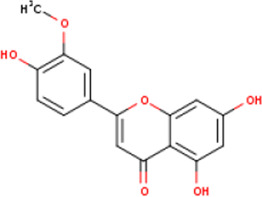	NOS2; CDK2; PPARG; PTGS2; MAPK14
MOL000422	Kaempferol	520-18-3	286.2	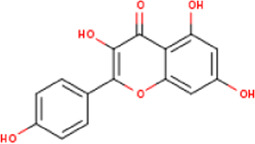	NOS2; NOS3; PPARG; PTGS2
MOL005093	Diosmin	520-27-4	608.5	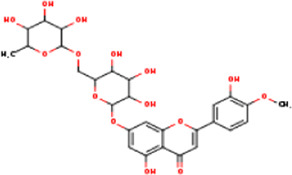	PTPN1; PTGS2
MOL005530	Hydroxygenkwanin	20243-59-8	300.2	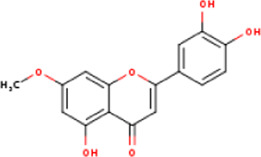	NOS2; PTGS2
MOL000006	Luteolin	491-70-3	286.2	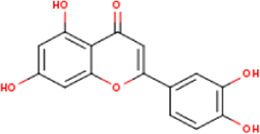	PTGS2
MOL000748	HMF	67-47-0	126.1	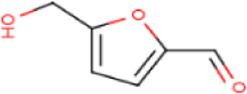	PTGS2
MOL000008	Apigenin	520-36-5	270.2	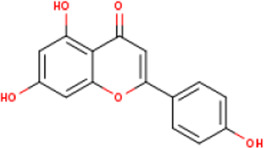	PTGS2
MOL000098	Quercetin	117-39-5	302.2	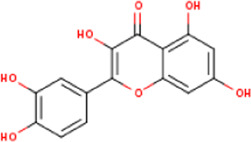	NOS3; MMP3; PPARG; PTGS2

### Molecular Docking of the Compounds With DPP4

**FIGURE 4:**
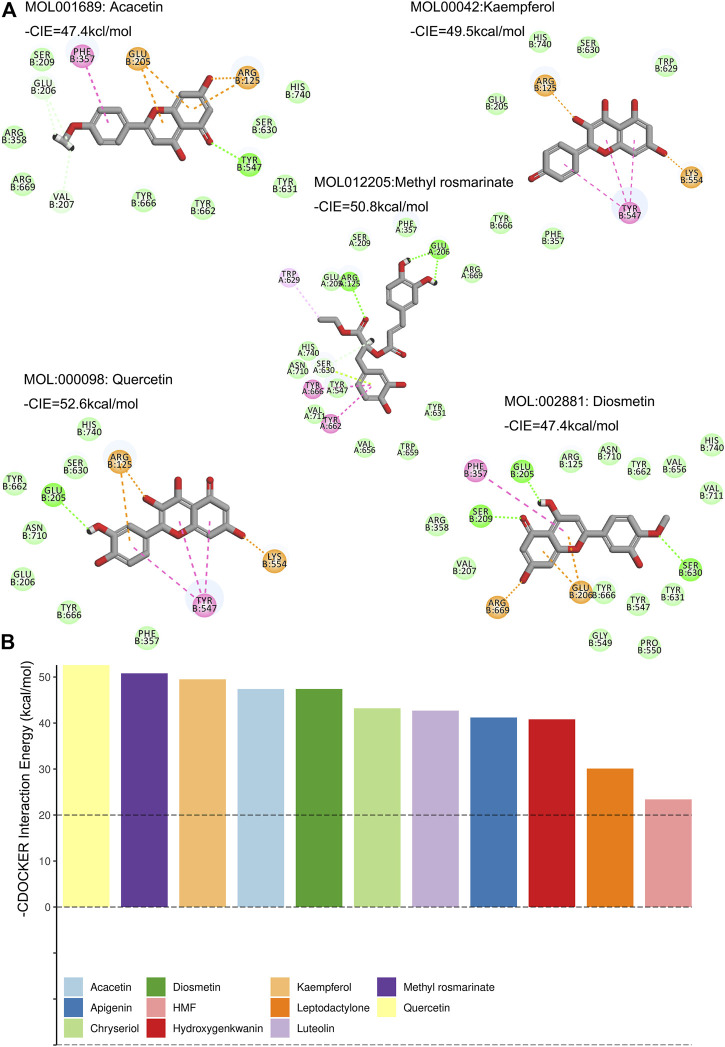
Molecular docking of compounds and DPP4. **(A)** Molecular docking between the compounds and DPP4 (Show only—CIE top five ranked). Green represents hydrogen bonding, pink represents PI-PI stacked, gold represents salt bridge, green amino acids at the periphery for van der Waals forces. **(B)** These compounds were ranked according to their -CIE with DPP4. The top five compounds were Quercetin, Methyl rosmarinate, Kaempferol Diosmetin and Acacetin.

In our study, DPP4 was arranged to dock with the 12 compounds. Except for Diosmin, the remaining 11 compounds can be docked with DPP4 via the DS software (Figure 4A, Supplementary Figure 3). The top five small molecule compounds that interacted with DPP4 were Quercetin, Methyl rosmarinate, Kaempferol Diosmetin and Acacetin. The -CDOCKER Interaction Energy (CIE) of DPP4 and the above active ingredients was 52.6, 50.6, 49.5, 47.4, and 47.4 kcal/mol (Figure 4B). According to previous publications on drug-protein interactions, compounds with -CIE of approximately 50 kcal/mol can be used as effect moles for their putative targets (Li et al., 2013; Jin et al., 2017; Yan et al., 2019).

### Inhibition of DPP4 Activity And Cell Growth In Human Immortalized Podocytes By The Active Ingredient of The LCH

**FIGURE 5:**
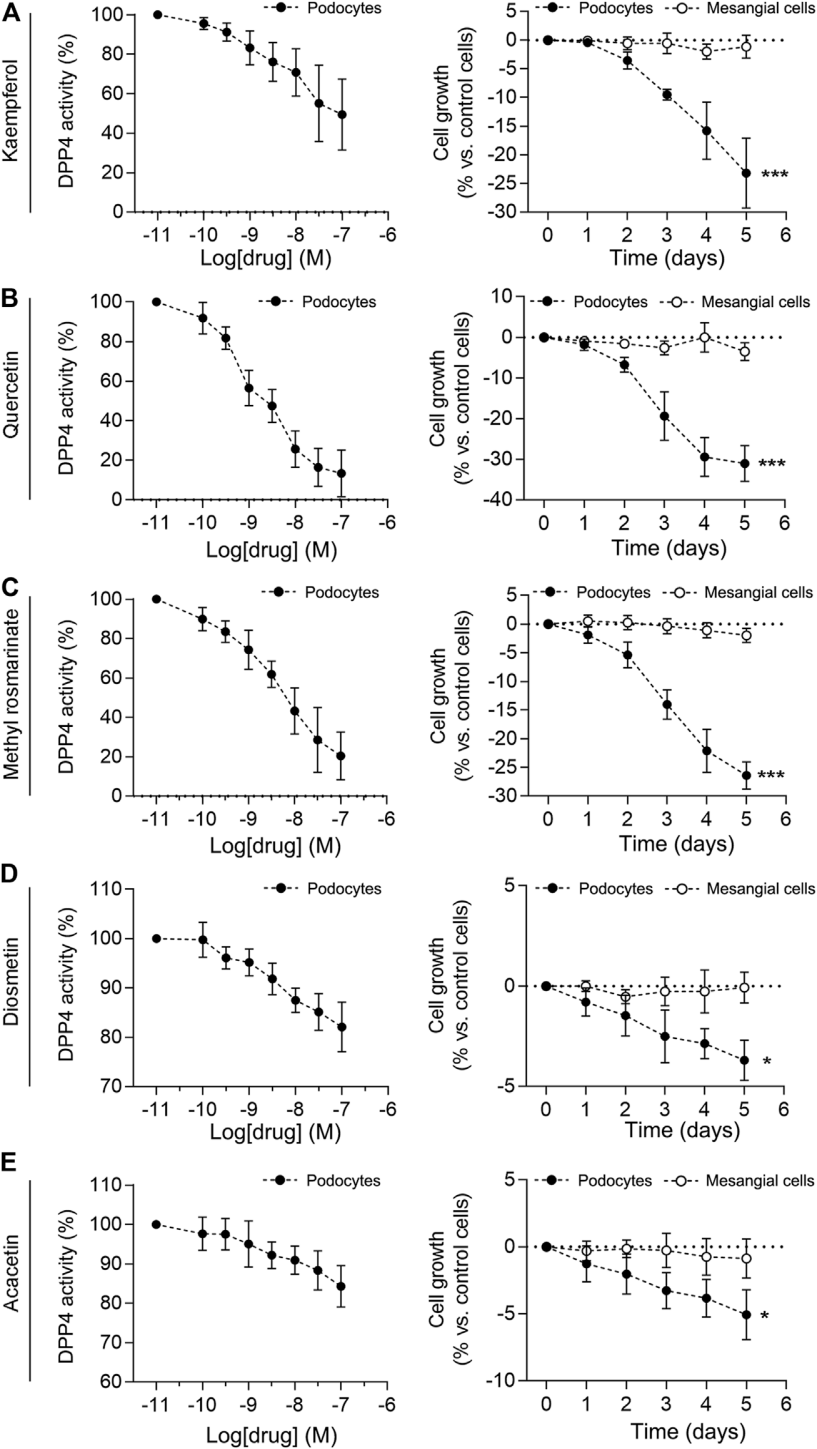
Effect of compounds on DPP4 activity and cell growth in immortalized podocytes and mesangial cells. **(A–E)** Effects of increasing compounds concentrations (0.01–100 nM) on the enzymatic activity measured in immortalized human podocytes were shown in the left panel. Enzymatic activity was evaluated in cell extracts by measuring the rate of increase in fluorescence intensity, expressed as arbitrary units (A.U.)·min^−1^, normalized to protein content. To set the *Y*-axis, all data were normalized to the mean value of the control group. Immortalized human podocytes and mesangial cells were exposed to either vehicle alone (control) or compounds for 5 days, and cell growth was measured by a colourimetric assay in the right panel. *n* = 3. *p < 0.05, ***p < 0.001 vs mesangiaal cells, by unpaired Student’s *t*-test.

To determine the effect of the active constituents of LCH on DPP4, we determined the activity of DPP4 in podocytes at different compounds. The results showed that Quercetin, Methyl rosmarinate, Kaempferol, Diosmetin and Acacetin significantly inhibited the activity of DPP4 in a concentration-dependent manner (Figures 5A–E). In contrast, the activity of DPP4 in mesangial cells used as a control was not inhibited by these components. Previous studies have found that in the DKD model, DPP4 not only increases significantly in podocyte expression but also promotes podocyte proliferation (Miglio et al., 2017). Further, the proliferation of podocytes promoted the development of glomerular disease (Ding et al., 2006; Rizzo et al., 2013). Specific evidence of podocyte proliferation was observed in DKD mice, as evidenced by increased staining for the proliferation markers PCNA and Ki67 in glomerular podocytes (Herman-Edelstein et al., 2011). We exposed the mesangial cells to each of the five compounds for 5 days and found no significant change in cell growth. Interestingly, when podocytes were exposed to these compounds, a decrease in cell growth was observed on day 1 or 2 and was positively correlated with time, with the most significant growth inhibition occurring on day 5 (Figures 5A–E). These results suggest that Quercetin, Methyl rosmarinate, Kaempferol, Diosmetin and Acacetin may retard the growth of podocytes through the inhibitory effect of DPP4.

### Effect of the Active Ingredients of the LCH on the Cell Cycle of Human Immortalized Podocytes

**FIGURE 6:**
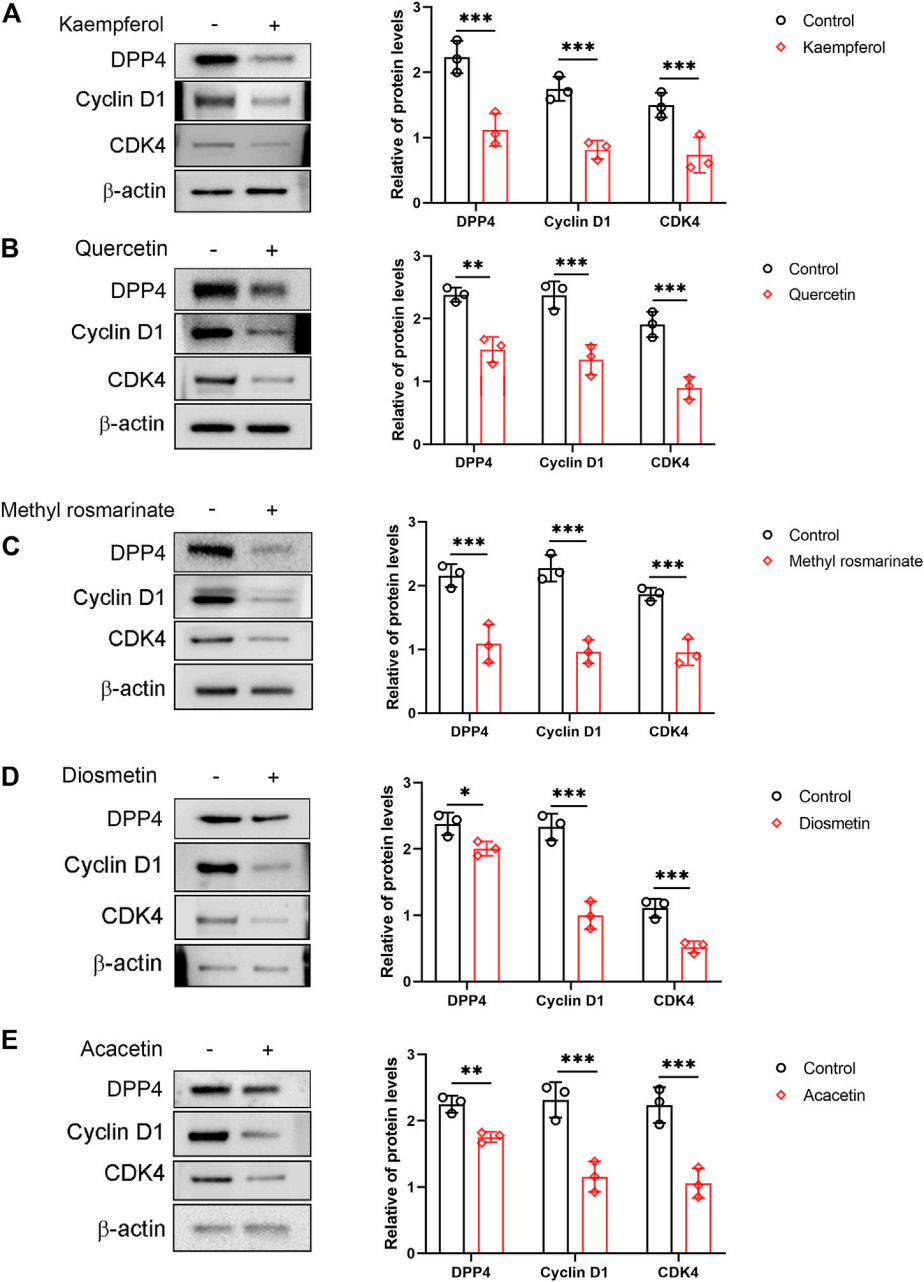
Effects of compounds on the expression of cyclin D1 and CDK4 in immortalized human podocytes. **(A–E)** Immortalized human podocytes were exposed to either vehicle alone (control) or compounds (10 nM) for 5 days, then the expression of DPP4, cyclin D1 and CDK4 was evaluated by western blot analyses. β-Actin was adopted as an internal standard to control for unwanted sources of variation. *n* = 3. *p < 0.05, **p < 0.01, ***p < 0.001 vs normal control, by unpaired Student’s *t*-test.

The proliferation of podocytes is associated with an abnormal cell cycle. This effect can be reflected by cell cycle-related proteins, including Cyclin D1 and CDK4. In the present study, a western blot was used to detect the expression of Cyclin D1 and CDK4. The expression of Cyclin D1 and CDK4 was significantly reduced in cells treated with Quercetin, Methyl rosmarinate, Kaempferol, Diosmetin and Acacetin compared to control cells (Figures 6A–E). In addition, we also found that the protein level of DPP4 was significantly decreased in cells treated with Quercetin, Methyl rosmarinate, Kaempferol, Diosmetin and Acacetin compared to control cells (Figures 6A–E).

### Druglikeness and Pharmacokinetics Assay of the Active Ingredients of LCH

**FIGURE 7:**
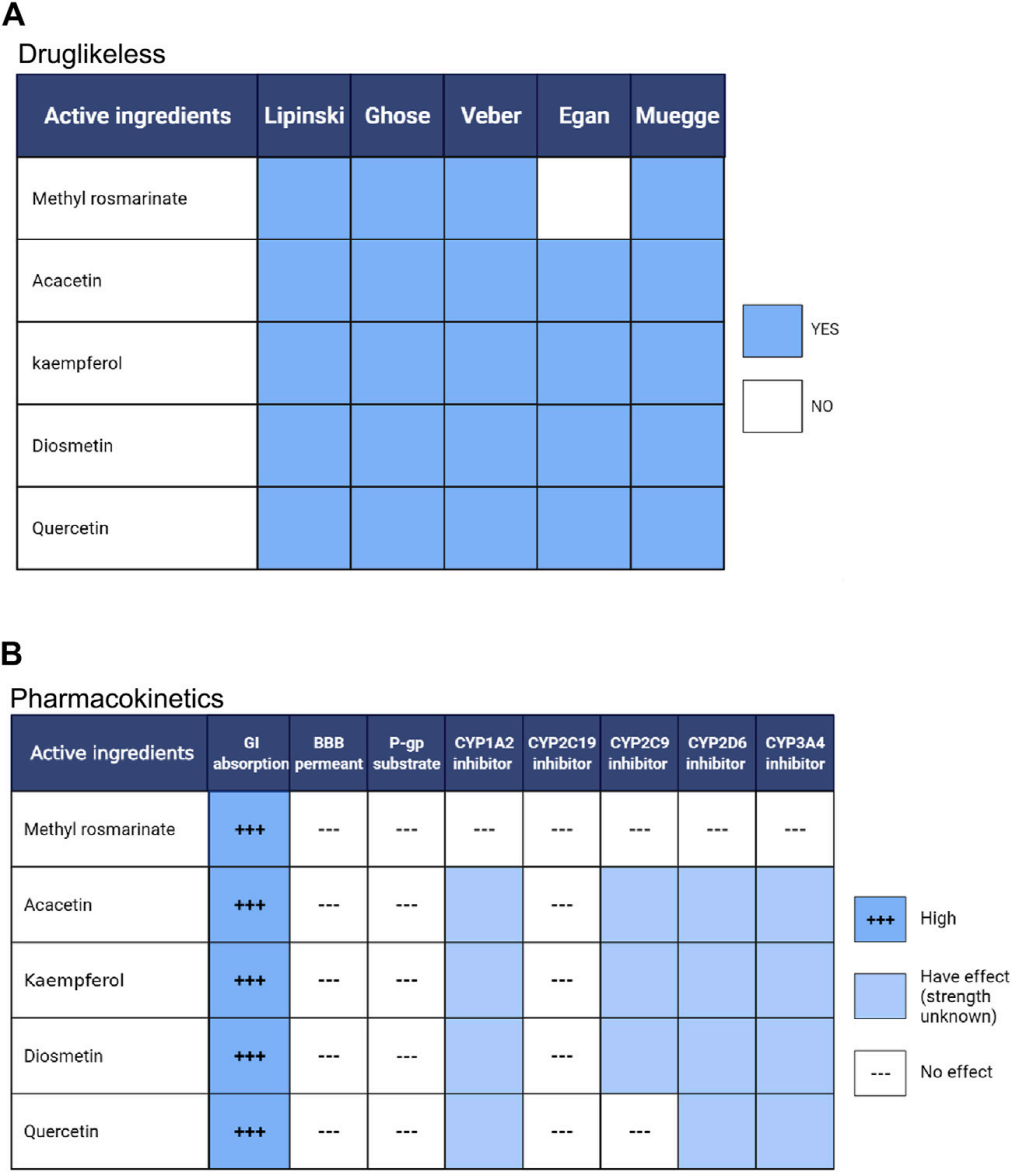
Pharmacokinetic **(A)** and drug-likeness **(B)** analysis of compounds. GI absorption, Gastrointestinal absorption; BBB permeant, blood-brain barrier permeant; P-gp substrate, permeability glycoproteins substrate, CYP, cytochrome p450. Created by Biorender(https://biorender.com/).

To clarify the drug-like properties of Quercetin, Methyl rosmarinate, Kaempferol, Diosmetin and Acacetin, they were filtered separately using the Lipinski, Ghose, Veber, Egan and Muegge methods (Figure 7A). As a consequence, these compounds were found to satisfy most of the filtration conditions, indicating that they have excellent drug-like properties. By pharmacokinetic analysis, all the five compounds were efficiently absorbed by the gastric and intestinal systems (Figure 7B). In addition, none of them was able to cross the blood-brain barrier. Kaempferol, Acacetin and Diosmetin are inhibitors of CYP1A2, CYP2C9, CYP2D6 and CYP3A4. Quercetin is also an inhibitor of CYP1A2, CYP2D6 and CYP3A4. Therefore, these compounds should be avoided in combination with drugs requiring the metabolism of these CYP isomers, whereas Methyl rosmarinate has no such restrictions. Quercetin, Methyl rosmarinate, Kaempferol, Diosmetin and Acacetin were determined to have excellent drug-likeness and pharmacokinetic properties by the above analysis.

### Quercetin Inhibited Abnormal Proliferation of Podocytes and DPP4 Activitiy in the Kidney of *db/db* Mice

Quercetin (50 mg/kg body weight) was given daily by oral gavage beginning from 8 week-old *db/db* mice for 12 weeks. After treatment, mice were killed, and kidneys were collected. According to our results, WT1 (podocyte marker) positive cells decreased in the kidney of *db/db* mice compare with C57BLKS/J mice, while Quercetin treatment significantly increased the WT1 positive cells in the kidney of *db/db* mice (Supplementary Figures S4A,B). In addition, when compared with C57BLKS/J mice, Ki67 and WT1 positive cells were increased in the kidney of mice, which suggest that there was abnormal proliferation of podocytes in the kidney of *db/db* mice (Supplementary Figures S4A,B). After Quercetin intervention, Ki67 and WT1 positive cells were decreased in the kidney of *db/db* mice. Furthermore, DPP4 activity was upregulated in the kidney of *db/db* mice when compare with C57BLKS/J mice, and Quercetin intervention decreased DPP4 activity in the kidney of *db/db* mice (Supplementary Figure S4C).

## Discussion

DKD is a complex disease that associates multiple proteins or pathways in its development and progression. DPP4 inhibitors, an emerging drug for diabetes treatment, have been found to exhibit renoprotective effects in addition to glucose-lowering effects (Hasan and Hocher, 2017; Nicotera et al., 2020). In the present study, the active ingredient of LCH was potentially found to treat DKD by inhibiting DPP4.

Analysis of single-cell sequencing data from mouse kidneys showed that not only did podocytes proliferation occur in DKD glomeruli, but DPP4 expression in podocytes was also specifically upregulated. Previous studies have discovered that DPP4 inhibits the proliferation of podocytes, hence we hypothesize that the active ingredient of LCH retards the proliferation of podocytes by inhibiting the expression of DPP4 (Miglio et al., 2017). We also discovered that DPP4 caused podocyte proliferation in DKD mice in conjunction with other proteins. An interaction exists between DPP4 and a fraction of genes that were differentially expressed in podocytes and most significantly enriched in the FSGS signalling pathway. Notably, FSGS is a disease characterised by podocytes damage (Fogo, 2015). The significance of the target gene of an active compound enriched in the DKD signalling pathway provides a method of drug screening. Twelve active constituents of LCH with DPP4 as the target gene were screened through the TCSMP database. Subsequent enrichment analysis of the signalling pathways of each of these 12 compounds for target genes excluding DPP4 revealed that Apigenin, HMF and Luteolin were not significantly enriched in DKD-related pathways. Interestingly, in subsequent experiments, it was also demonstrated that these three compounds did not affect DPP4 activity or the growth of podocytes. Molecular docking is a hugely effective drug screening technique (Morris and Lim-Wilby, 2008). In the current study, 12 compounds that were initially screened through the TCSMP database for possible DPP4 targets were used for docking with DPP4. The top five compounds in terms of their ability to inhibit DPP4 activity were Quercetin, Methyl rosmarinate, Kaempferol, Diosmetin and Acacetin, which were shown in subsequent experiments to inhibit the activity of DPP4 in podocytes.

Single-cell sequencing of mouse DKD kidneys suggests a positive correlation between upregulation of DPP4 and podocyte proliferation. Notably, DPP4 inhibitors have been demonstrated to reduce the growth rate of different cell types (Schön et al., 1985; Reinhold et al., 1997). Based on the above results, we designed the following experiments: podocytes were exposed to the active ingredients of each of the 12 compounds to observe whether the activity of DPP4 and the growth rate of podocytes would be affected. Quercetin, Methyl rosmarinate, Kaempferol, Diosmetin and Acacetin treated podocytes exhibited positive results and were found to prolong the cell cycle by inhibiting the expression of Cyclin D1 and CDK4. *In vivo*, our results showed that there is abnormal proliferation in the podocyte of *db/db* mice (Supplementary Figures S4A,B). Consistent with this, previous study has also proved that podocyte abnormal proliferation was observed in DKD mice (Herman-Edelstein et al., 2011). Importantly, Quercetin intervention could inhibt the DPP4 activity in the kidney of *db/db* mice (Supplementary Figure S4C). These results indicated that the active component of LCH not only inhibited the activity of DPP4 in podocytes, but also suppressed the proliferation of podocytes and the expression of cell cycle proteins.

Quercetin, Methyl rosmarinate, Kaempferol Diosmetin and Acacetin have been demonstrated *in vitro* to suppress the proliferation of podocytes by inhibiting DPP4 and have potential as therapeutic agents for DKD. To be an effective drug, however, an effective molecule must reach its target in the body with a sufficient concentration and remain there long enough in its biologically active form (Daina et al., 2017b). Hence, pharmacokinetics and drug-likeness evaluations of these compounds are mandatory. “Drug likeness” assesses the probability of a molecule becoming an orally administered drug. The SwissADME online analysis tool offers filters based on five different rules for the screening of drug-like. Quercetin, Methyl rosmarinate, Kaempferol, Diosmetin and Acacetin satisfy most of the drug-like criteria and have a high probability of becoming oral drugs. Compound pharmacokinetics are influenced by GI absorption, BBB permeation, P-gp substrates and CYP inhibitors. The GI absorption of Quercetin, Methyl rosmarinate, Kaempferol, Diosmetin and Acacetin was high, indicating excellent oral bioavailability. P-gp is the most important member of the ATP binding cassette transporter or ABC transporter (Desai et al., 2012). Its main role relates to the protection of the central nervous system from the effects of drugs as well as the promotion of drug resistance (Szakács et al., 2008; Montanari and Ecker, 2015). None of these five compounds is substrates of P-gp and does not affect drug resistance or the central system.

Notably, contrary to previous findings, the present study revealed that the podocytes in DKD mice were proliferating. The single-cell sequencing analysis was performed on streptozotocin-induced 10-week-old mice with kidney lesions in the early stages of DKD. We speculate that podocyte proliferation and apoptosis are dynamic in the development of DKD. An inability to observe podocyte proliferation in DKD is a misconception. Podocyte proliferation was readily observed in experimental models of selective glomerular injury, as some podocytes re-engaged in the cell cycle as an adaptive response to injury (Macconi et al., 2009). Herman-Edelstein et al. first observed podocyte proliferation in DKD glomeruli (Herman-Edelstein et al., 2011). In the early stages of DKD, abnormal podocyte proliferation leads to cellular and collapsed hyperplasia, while in the late stages of DKD, abnormal apoptosis leads to podocytopenia and segmental glomerulosclerosis by exposing the basement membrane to form adhesions (Herman-Edelstein et al., 2011). In summary, we assume that aberrant proliferation is the initiating manifestation of DKD podocyte injury, which disrupts the dynamic balance between apoptosis and proliferation. The active ingredients of LCH can slow down the progression of the disease by inhibiting the proliferation of podocytes in the early stages of DKD.

Both Quercetin and Kaempferol have been reported in clinical study dosages. An intake dose of 150–500 mg/day of Quercetin is considered safe (U.S. Food and and Drug Administration, 2018; Egert et al., 2009). Daily intake of 8.04 mg/day of Kaempferol produced beneficial effects with no reported adverse events (Bobe et al., 2010; Bai et al., 2014). Although there are no clinical studies on Methyl rosmarinate, Diosmetin and Acacetin, their doses have been reported in animal studies. Methyl rosmarinate (50 mg/kg) was found to have anti-hyperglycaemic and anti-diabetic activity in mice and no toxicity was observed (Giles-Rivas et al., 2020). Experimental concentration of Diosmetin 20 μM attenuated acute kidney injury in mice (Wang et al., 2020). Acacetin reduces blood glucose levels in streptozotocin (STZ)-induced diabetic mice at a dose of 31.6 mg/kg (Singh et al., 2020). However, the optimal concentration of these drugs to treat patients with DKD still needs to be verified by a large number of experiments

## Conclusion

In conclusion, a combination of single-cell sequencing, network pharmacology prediction and experimental validation was applied to explore the pharmacological mechanism of action of LCH in the treatment of DKD. Single-cell sequencing data revealed that DPP4 was specifically upregulated in the podocytes of DKD mice and was associated with podocyte proliferation. Further network pharmacological predictions and experimental validation studies revealed that Quercetin, Methyl rosmarinate, Kaempferol, Diosmetin and Acacetin were effective small molecule drugs that inhibited the expression of DPP4 and delayed the proliferation of podocytes for the treatment of DKD. Future *in vivo* trials could be conducted to explore the potential efficacy of these LCH active ingredients in the treatment of DKD.

## Data Availability

Publicly available datasets were analyzed in this study. This data can be found here: The Single-cell profiling of kidney cells sequencing data was acquired from the Gene Expression Omnibus (GEO) GSE127235 dataset of the National Center for Biotechnology Information (NCBI; https://www.ncbi.nlm.nih.gov/gds).
