# Supplementary material for: Inhibition of Podocytes DPP4 Activity Is a Potential Mechanism of Lobeliae Chinensis Herba in Treating Diabetic Kidney Disease
Source: Front Pharmacol. 2021 Dec 7;12:779652. doi: 10.3389/fphar.2021.779652 (PMC8688925; doi:10.3389/fphar.2021.779652)
Supplement: Supplementary file 1 [file Presentation1.pdf]

Supplement Figure for

Inhibition of Podocytes DPP4 Activity is a Potential Mechanism of Lobeliae Chinensis Herba in Treating Diabetic kidney Disease

Xinyu Wang[1,#], Jiaqing Xiang[1,#], Guixiao Huang[2,#], Lin Kang[1], Guangyan Yang[1], Han Wu[3], Kewei Jiang[1], Zhen Liang[1,*], Shu Yang[1,*]

[1]Department of Geriatrics, The Second Clinical Medical College of Jinan University, Shenzhen People’s Hospital, Jinan University, Shenzhen, China

[2]The 3rd Affiliated Hospital of Shenzhen University, Shenzhen, China

[3]Department of Endocrinology, The Second Clinical Medical College of Jinan University, Shenzhen People’s Hospital, Jinan University, Shenzhen, China

Supplement Methods

In vivo studies with animals

All experimental protocols for animal care and in vivo studies conform to the Guide for the Care and Use of Laboratory Animals published by the National Institutes of Health (NIH) (NIH Publication No. 85-23, revised 1996). ARRIVE guidelines are the standard to follow for animal research reports (1). The Ethics Committee of the Second School of Clinical Medicine of Jinan University (Shenzhen People's Hospital) approved the animal study. Male type 2 diabetic (db/db) and C57BLKS/J wild-type mice (8 weeks old) were purchased from Nanjing University Animal Centre (Nanjing, Jiangsu, China). Mice were accommodated in the SPF unit (12-hour light cycle from 8am to 8pm, 23±1°C, 60-70% humidity) at the Shenzhen People's Hospital Animal Centre, maintained on standard rodent food and with free access to water in plastic bottles. The mice were kept in plastic cages lined with maize bedding. The amount of mice per cage did not exceed 5. Quercetin (50mg/kg body weight) was given daily by oral gavage beginning from 8 week-old db/db mice for 12 weeks. All mice were anaesthetised and euthanised in a CO_2 chamber before liver and blood samples were collected.

Immunofluorescence and Western Blotting

Cells were grown on coverslips, washed twice with PBS, fixed in 4% paraformaldehyde for 20 minutes, permeabilised with 1% SDS and incubated in blocking buffer (1% BSA, 0.25% Triton 3100, pH 7.4). The primary and secondary antibodies were diluted in blocking buffer and the cells with antibodies were incubated overnight at 4°C. A coverslip containing Prolog Gold Antifade Reagent (Invitrogen, Carlsbad CA) with DAPI was then mounted onto a glass microscope slide. The main antibodies used include marker of proliferation Ki-67 and WT1. Western blotting experiments were performed using DPP4, Cyclin D1 and CDK4 antibodies, as previously described (2).

Supplement Figure 1

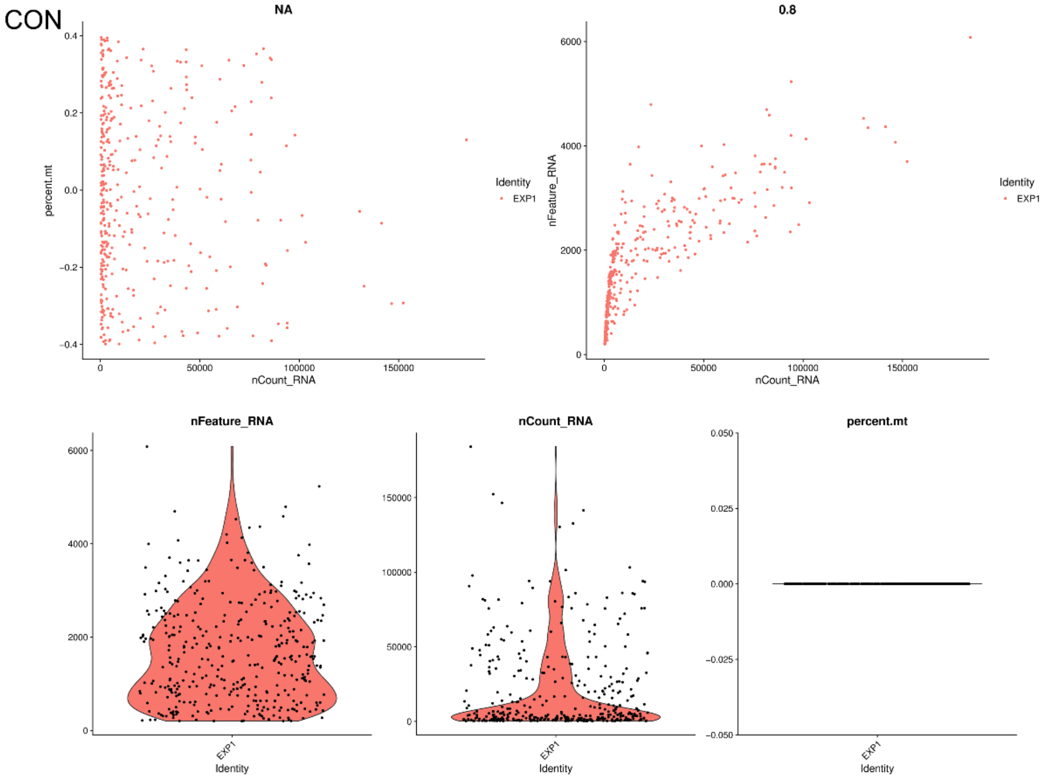

Supplement Figure 1. Control group quality control of single-cell sequencing data. nFeature_RNA represents the number of genes measured per cell, nCount represents the sum of the expression of all genes measured per cell, and percent.mt represents the proportion of mitochondrial genes detected per cell.

Supplement Figure 2

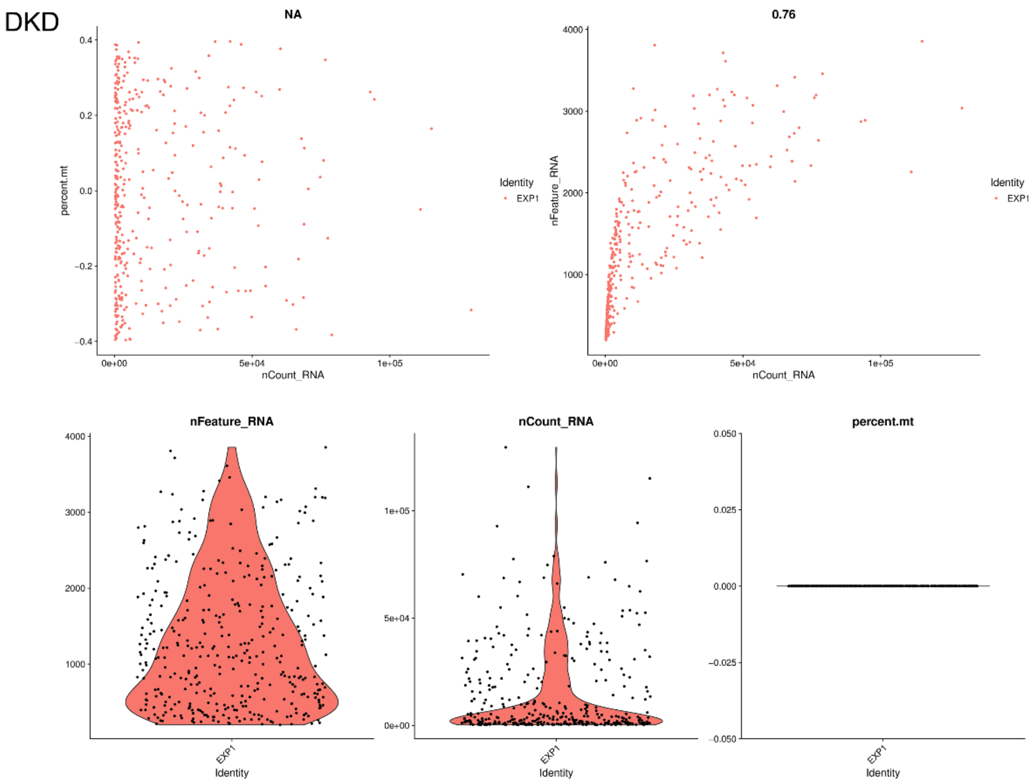

Supplement Figure 2. DKD quality control of single-cell sequencing data. nFeature_RNA represents the number of genes measured per cell, nCount represents the sum of the expression of all genes measured per cell, and percent.mt represents the proportion of mitochondrial genes detected per cell.

Supplement Figure 3

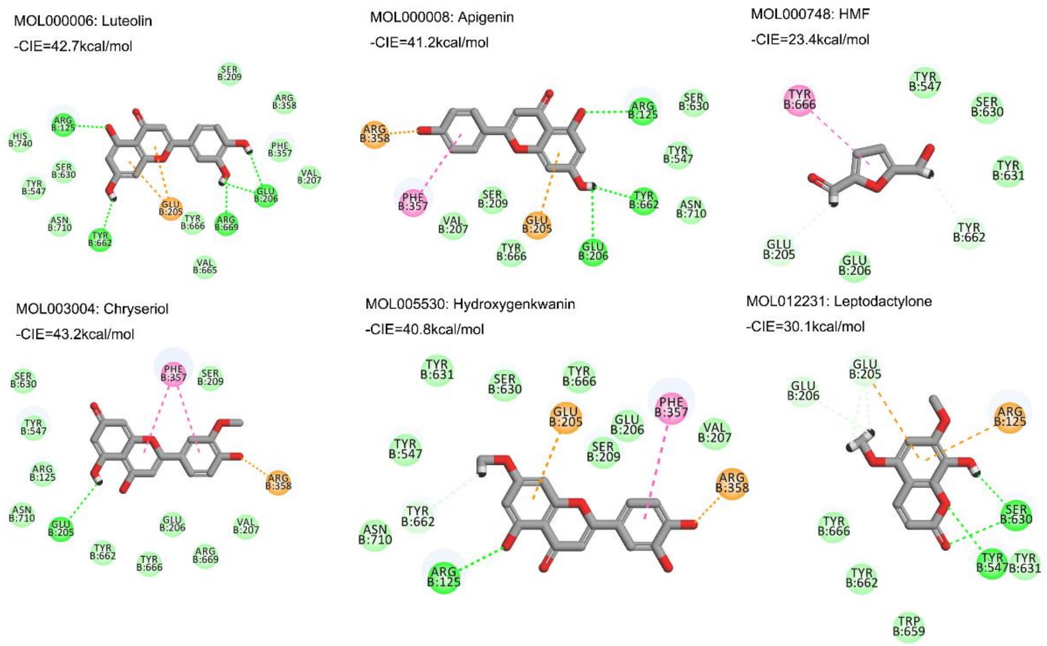

Supplement Figure 3. Molecular docking of compounds and DPP4. Molecular docking between the compounds and DPP4 (Show only - CIE ranked 6th to 11th, Diosmin cannot be docked with DPP4). Green represents hydrogen bonding, pink represents PI-PI stacked, gold represents salt bridge, and red represents unfavourable donor-donor/acceptor-acceptor, green amino acids at the periphery for van der Waals forces.

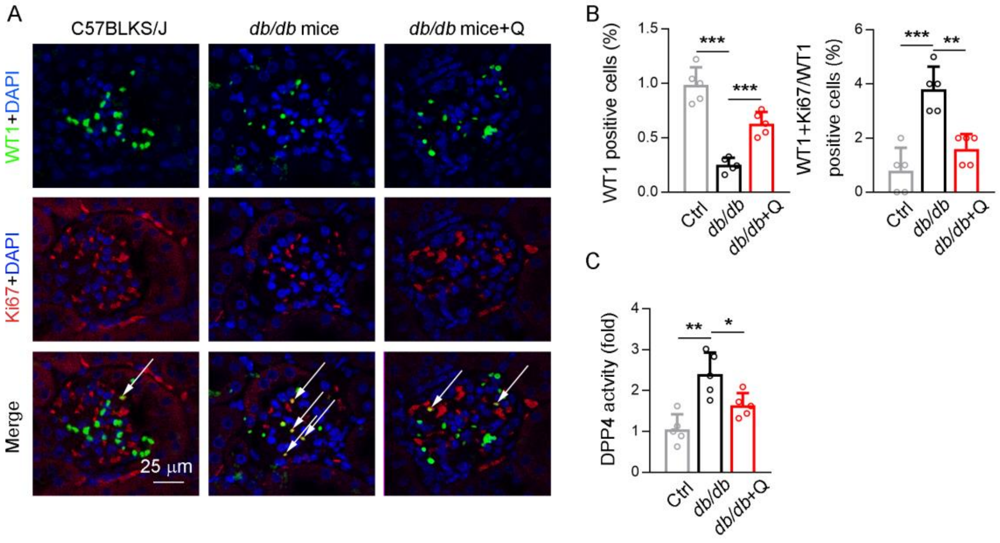

Supplement Figure 4. Quercetin treatment inhibited the abnormal proliferation of podocyte and DPP4 activity in the kidney of *db/db* mice. (A-C) Quercetin (50 mg/kg body weight) was given daily by oral gavage beginning from 8 week-old db/db mice for 12 weeks. Representative images of WT and Ki67 immunofluorescence were shown (A). Quantitative results of panel A were counted in the kidney in 3 fields per animal at×100 magnification (B). The DPP4 activity was detected by kit in the kidney of *db/db* mice, n=5 (C).*$p<0.05$, **$p<0.01$,***$p<0.001$, by One-way ANOVA.

1. McGrath JC, Lilley E. Implementing guidelines on reporting research using animals (ARRIVE etc.): new requirements for publication in BJP. Br J Pharmacol. 2015;172(13):3189-3193.
2. Yang S, Ma C, Wu H, Zhang H, Yuan F, Yang G, Yang Q, Jia L, Liang Z, Kang L. Tectorigenin attenuates diabetic nephropathy by improving vascular endothelium dysfunction through activating AdipoR1/2 pathway. Pharmacol Res. 2020;153:104678.
